# *Protochlamydia* Induces Apoptosis of Human HEp-2 Cells through Mitochondrial Dysfunction Mediated by Chlamydial Protease-Like Activity Factor

**DOI:** 10.1371/journal.pone.0056005

**Published:** 2013-02-11

**Authors:** Junji Matsuo, Shinji Nakamura, Atsushi Ito, Tomohiro Yamazaki, Kasumi Ishida, Yasuhiro Hayashi, Mitsutaka Yoshida, Kaori Takahashi, Tsuyoshi Sekizuka, Fumihiko Takeuchi, Makoto Kuroda, Hiroki Nagai, Kyoko Hayashida, Chihiro Sugimoto, Hiroyuki Yamaguchi

**Affiliations:** 1 Department of Medical Laboratory Science, Faculty of Health Sciences, Hokkaido University, Sapporo, Hokkaido, Japan; 2 Division of Biomedical Imaging Research, Juntendo University Graduate School of Medicine, Tokyo, Japan; 3 Division of Ultrastructural Research, Juntendo University Graduate School of Medicine, Tokyo, Japan; 4 Pathogen Genomics Center, National Institute of Infectious Diseases, Shinjuku-ku, Tokyo, Japan; 5 Research Institute for Microbial Diseases, Osaka University, Suita, Osaka, Japan; 6 Research Center for Zoonosis Control, Hokkaido University, Kita-ku, Sapporo, Japan; University of California Merced, United States of America

## Abstract

Obligate amoebal endosymbiotic bacterium *Protochlamydia* with ancestral pathogenic chlamydial features evolved to survive within protist hosts, such as *Acanthamoba*, 0.7–1.4 billion years ago, but not within vertebrates including humans. This observation raises the possibility that interactions between *Protochlamydia* and human cells may result in a novel cytopathic effect, leading to new insights into host-parasite relationships. Previously, we reported that *Protochlamydia* induces apoptosis of the immortalized human cell line, HEp-2. In this study, we attempted to elucidate the molecular mechanism underlying this apoptosis. We first confirmed that, upon stimulation with the bacteria, poly (ADP-ribose) polymerase (PARP) was cleaved at an early stage in HEp-2 cells, which was dependent on the amount of bacteria. A pan-caspase inhibitor and both caspase-3 and -9 inhibitors similarly inhibited the apoptosis of HEp-2 cells. A decrease of the mitochondrial membrane potential was also confirmed. Furthermore, lactacystin, an inhibitor of chlamydial protease-like activity factor (CPAF), blocked the apoptosis. Cytochalasin D also inhibited the apoptosis, which was dependent on the drug concentration, indicating that bacterial entry into cells was required to induce apoptosis. Interestingly, *Yersinia* type III inhibitors (ME0052, ME0053, and ME0054) did not have any effect on the apoptosis. We also confirmed that the *Protochlamydia* used in this study possessed a homologue of the *cpaf* gene and that two critical residues, histidine-101 and serine-499 of *C. trachomatis* CPAF in the active center, were conserved. Thus, our results indicate that after entry, *Protochlamydia*-secreted CPAF induces mitochondrial dysfunction with a decrease of the membrane potential, followed by caspase-9, caspase-3 and PARP cleavages for apoptosis. More interestingly, because *C. trachomatis* infection can block the apoptosis, our finding implies unique features of CPAF between pathogenic and primitive chlamydiae.

## Introduction

Members of the order *Chlamydiales* are obligate intracellular bacteria that were discovered about a century ago. Although ancient chlamydiae diverged into pathogenic and primitive chlamydiae 0.7–1.4 billion years ago, all pathogenic chlamydiae species have co-evolved with their vertebrate hosts and so-called primitive chlamydiae have evolved as endosymbionts of lower eukaryotes, namely free-living amoebae (*Acanthamoeba*) complete with a powerful bacterial killing mechanism [1]–[4]. While pathogenic chlamydiae, including *Chlamydia trachomatis* and *C. pneumoniae*, are well-known human pathogens and the major cause of preventable blindness, as well as sexually transmitted and respiratory diseases [5]–[7], primitive chlamydiae, including *Parachlamydia acanthamoebae*, *Neochlamydia hartmanellae* and *Protochlamydia amoebophila*, are also likely to be implicated in human respiratory diseases and abortion [8]–[11]. Pathogenic chlamydiae have developed through a decrease in genome size and loss of redundant genes, which may be a strategy to evade the host immune network, resulting in a shift to parasitic energy and metabolic requirements, and genomes of approximately 1.0–1.2 Mb [2], [3]. However, the genome of representative primitive chlamydia (*Protochlamydia* UWE25) is not in the process of becoming smaller and has stabilized at 2.4 Mb [4]. This observation implies the possibility that, to overcome stressful conditions, primitive chlamydiae still possess certain molecules that pathogenic chlamydiae have already lost. Thus, comparison of the two chlamydiae, which have evolved separately through different paths and inhabiting niches, is extremely intriguing and may lead to new insights into host-parasite relationships.

The complicated manipulation mechanism of pathogenic chlamydiae, which occur in host cells, is becoming more obvious. It is the striking view that chlamydial type III effector proteins, which are inclusion membrane proteins (Incs), are primarily responsible for the process of inclusion biogenesis [12]–[16]. Furthermore, pathogenic chlamydiae also possess chlamydial protease-like activity factor (CPAF) that causes two significant modifications of cellular function [17]–[21]. One of the functions is responsible for inclusion maturation through cellular matrix degradation of the inclusion membrane backbone, thereby providing flexibility to mature inclusion bodies depending on the bacterial amounts [19]. More importantly, the other function contributes to prevention of apoptosis of infected cells through degradation of BH3-only proteins, which is a switch signal, followed by accumulation of Bax proteins, which induce pore formation on mitochondria, cytochrome *c* release, caspase-9 and -3 activation, and then poly (ADP-ribose) polymerase (PARP) cleavage for direct apoptosis induction [19], [20]. Furthermore, contrary to most T3SS effectors such as Incs, which exhibits little conservation at sequence level among chlamydial members, CPAF is a highly conserved protease, indicating a critical role to achieve pathogenic chlamydial survival in mammalian cells including human cells [17]–[21].

Thus far, in contrast to pathogenic chlamydiae, we have found an interesting feature of primitive chlamydia in which *Protochlamydia*, but not the killed bacteria, induce apoptosis of immortalized human HEp-2 cells [22], suggesting an unknown molecular mechanism of pathogenic chlamydial manipulation in cells. Therefore, we attempted to elucidate the molecular mechanism of primitive chlamydial apoptosis induction by either connecting type III effector or CPAF activities, which are critical regulators to achieve adaptation in mammalian cells.

## Results

### Apoptosis of HEp-2 Cells is Dependent on the Multiplicity of Infection (MOI) of *Protochlamydia* at an Early Stage

**Figure 1:**
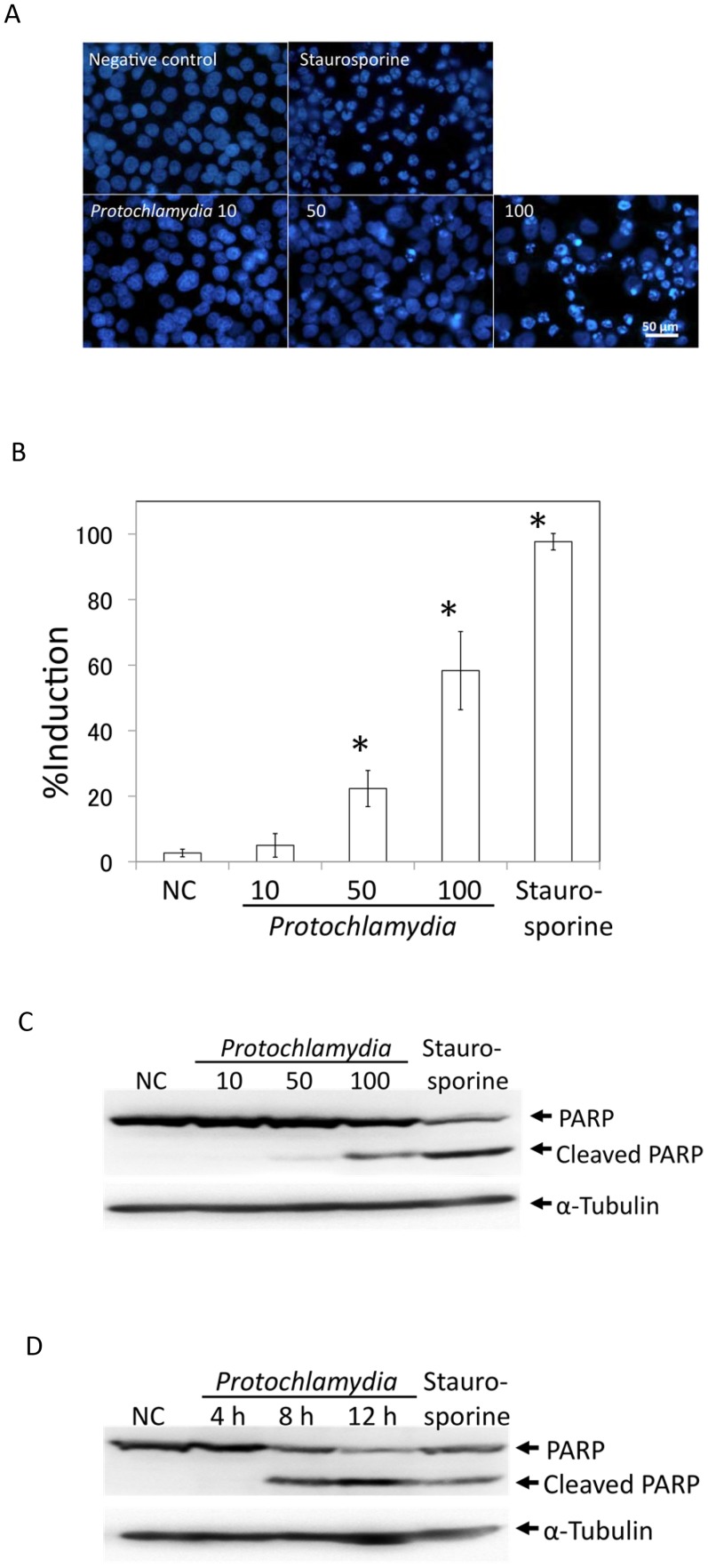
HEp-2 cell death induced by *Protochlamydia*. A) Representative images showing cell death of HEp-2 cells stimulated with bacteria at an MOI of 10, 50 and 100 at 24 h after incubation. Cell death was estimated by morphological nuclear changes as observed by DAPI staining under a fluorescence microscope. Typical morphological changes of segmented nuclei indicate cell death. Negative control; a culture without the bacteria. Staurosporine; a culture with the drug (10 µM). B) Numbers of dead cells in HEp-2 cell cultures induced by the addition of *Protochlamydia* and dependent on MOI. Cells were cultured with or without the bacteria adjusted to an MOI of 10–100 for up to 24 h. Staurosporine (10 µM); positive control. The number of dead cells was estimated by DAPI staining. Data are the means ± SD from at least three independent experiments performed in triplicate. *p<0.05 vs. without bacteria (NC). C) Representative western blot showing changes of PARP cleavage dependent on MOI. Cells stimulated with the bacteria for 8 h were collected and then subjected to western blotting with an antibody against PARP, an indicator of the apoptosis signaling cascade (See Methods). The presence of cleaved PARP indicates activation of the apoptosis pathway. α-tubulin was used for the internal control. NC; cells incubated without bacteria. Staurosporine (10 µM); positive control. D) Representative western blot showing changes of PARP cleavage dependent on incubation time. Cells stimulated with bacteria were collected at 4, 8 and 12 h after incubation and then subjected to western blotting with an antibody against PARP as mentioned above.

We first determined whether apoptosis induction was dependent on bacterial load or timing. As shown in Figure 1A and B, DAPI staining revealed that *Protochlamydia* obviously induced apoptosis of HEp-2 cells and, as expected, was dependent on bacterial MOI as demonstrated previously [22]. We also confirmed this feature by western blot analysis using PARP cleavage as a marker of apoptosis, which is located downstream of the apoptosis pathway [23], indicating maximum induction of apoptosis at an MOI of 100 (Figure 1C), possibly by the presence of unknown physical limitation on chlamydial adhesion to cells. We next determined the timing of HEp-2 cell apoptosis after incubation with the bacteria. As a result, PARP cleavage began at 8 h after incubation (Figure 1D). Taken together, the data revealed that some effector molecules might be involved in the apoptosis of HEp-2 cells.

### Apoptosis and Mitochondrial Dysfunction Followed by Caspase-9 and **−**3, and then PARP Cleavages

**Figure 2:**
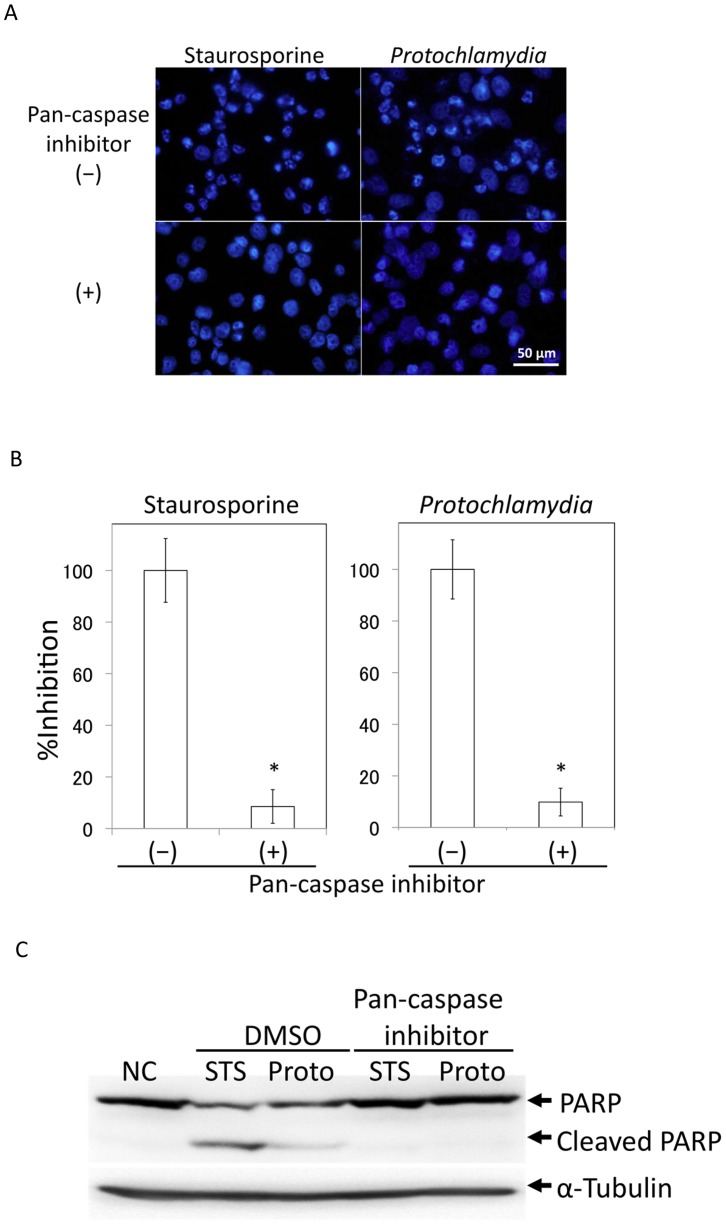
Effect of a pan-caspase inhibitor on *Protochlamydia*-induced apoptosis. A) Representative images showing cell death of HEp-2 cells stimulated with bacteria at an MOI of 100 in the presence or absence of a pan-caspase inhibitor (100 µM) at 24 h after incubation. Staurosporine (10 µM); positive control. B) Numbers of dead cells in HEp-2 cell cultures induced by the addition of *Protochlamydia* in the presence of a pan-caspase inhibitor. Cells were cultured with bacteria (MOI 100) or staurosporine (10 µM) in the presence or absence of a pan-caspase inhibitor (100 µM) for up to 24 h. The number of dead cells was estimated by DAPI staining. Data are the means ± SD from at least three independent experiments performed in triplicate. *p<0.05 vs. without the pan-caspase inhibitor (–). C) Representative western blot showing changes of PARP cleavage in the presence of a pan-caspase inhibitor. Cells stimulated with bacteria were collected at 8 h after incubation and then subjected to western blotting with an antibody against PARP.

**Figure 3:**
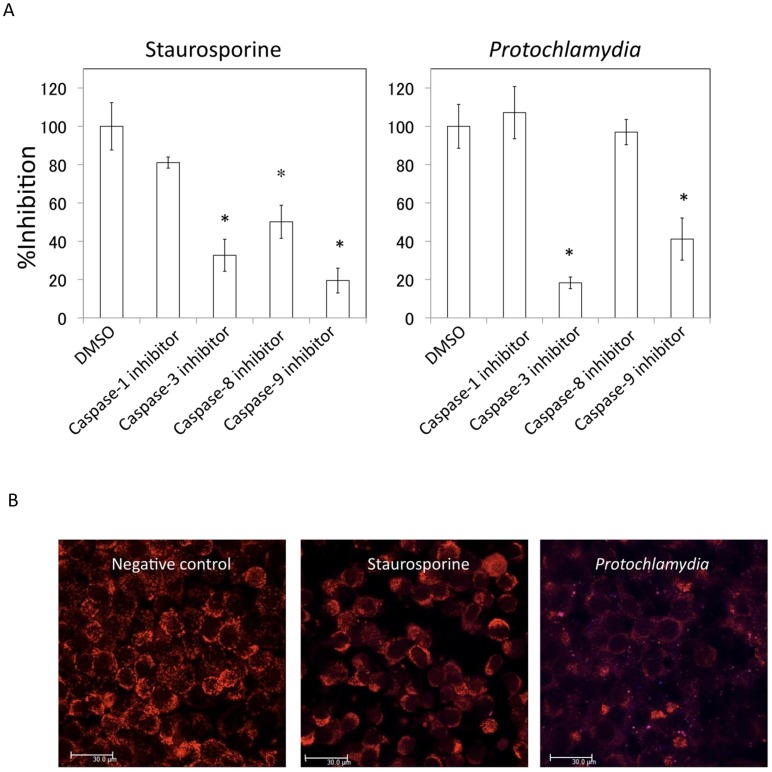
Both caspase −3 and −9 inhibitors block apoptosis with mitochondrial dysfunction. A) Effect of caspase inhibitors (caspase −1, −3, −8 and −9) on apoptosis. Numbers of dead cells in HEp-2 cell cultures induced by the addition of *Protochlamydia* in presence of each caspase inhibitor. Cells were cultured with bacteria (MOI 100) or staurosporine (10 µM) in the presence or absence of each caspase inhibitors (100 µM) for up to 24 h. The number of dead cells was estimated by DAPI staining. Data are the means ± SD from at least three independent experiments performed in triplicate. *p<0.05 vs. the absence of a caspase inhibitor (DMSO). B) A decrease of mitochondrial membrane integrity was observed in HEp-2 cells incubated with *Protochlamydia*. The integrity was assessed by a staining method using MitoTracker Red CMXRos (See Methods). Normal mitochondria are strongly stained as red (Negative control) compared with abnormal mitochondria (Staurosporine and *Protochlamydia*).

Pathogenic chlamydial CPAF directly contributes to the prevention of apoptosis of infected cells through degradation of BH3-only proteins to maintain infected host cells [19]–[21], which is a possible evolutionary path of pathogenic chlamydiae, revolving mitochondrial dysfunction. We therefore determined whether *Protochlamydia* could modulate mitochondrial function with activation of caspases and PARP cleavages. Using a DAPI staining assay, we found that a pan-caspase inhibitor obviously blocked *Protochlamydia*-induced apoptosis, and staurosporine, a stimulator that induces caspase-dependent apoptosis (Figure 2A and B). It was also confirmed by western blotting that the inhibitor blocked PARP cleavage (Figure 2C). Using several specific caspase inhibitors, we next determined which caspase molecule was involved in the induction of apoptosis. The results clearly indicated that caspase-3 and −9 inhibitors, but not caspase-1 and −8 inhibitors, blocked the apoptosis (Figure 3A), suggesting that apoptosis occurred through the mitochondrial pathway and was triggered by mitochondrial dysfunction. We also confirmed a decrease of the mitochondrial membrane potential in HEp-2 cells incubated with *Protochlamydia* (Figure 3B), suggesting mitochondrial dysfunction. Thus, taken together, we clearly observed that *Protochlamydia* induces apoptosis by mitochondrial dysfunction followed by activations of caspase-9 and −3, and PARP cleavage.

### Bacterial Entry into Cells is Required to Induce Apoptosis

**Figure 4:**
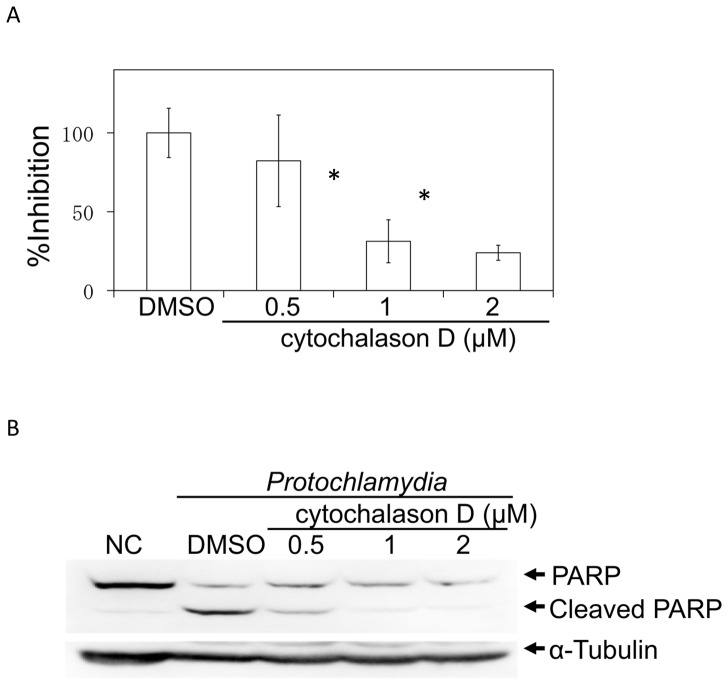
Effect of cytochalasin D on the induction of apoptosis. A) Numbers of dead cells in HEp-2 cell cultures induced by the addition of *Protochlamydia* (MOI 100) were estimated in the presence or absence of cytochalasin D (0.5–2 µM). Cells were cultured with or without bacteria for up to 24 h. The number of dead cells was estimated by DAPI staining. Data are the means ± SD from at least three independent experiments performed in triplicate. *p<0.05 vs. DMSO. B) Representative western blot showing changes of PARP cleavage in the presence of cytochalasin D (0.5–2 µM). DMSO; control. Cells stimulated with bacteria were collected at 8 h after incubation and then subjected to western blotting with an antibody against PARP.

We assessed whether the apoptosis induced by *Protochlamydia* was required for bacterial entry into cells using cytochalasin D, an inhibitor that blocks actin remodeling. As a result, the number of dead cells was significantly decreased by treatment with cytochalasin D, which was dependent on the drug concentration (Figure 4A). We also confirmed that the amount of cleaved PARP was clearly decreased by cytochalasin D treatment (Figure 4B). Taken together, the results indicated that bacterial entry into cells is required to induce apoptosis on HEp-2 cells, suggesting that effector molecules secreted into the cytoplasm by bacteria may be involved in the apoptosis.

### *Yersinia* Type III Effector Inhibitors do not Prevent *Protochlamydia*-induced Apoptosis

Genome sequencing revealed genes encoding putative type III machinery in the genomes of primitive (*Protochlamydia* UWE25) [4] and pathogenic chlamydiae [13], [14]. Interestingly, it appears likely that the features of the chlamydial type III gene cluster are closely related to those of *Yersinia* type III gene clusters based on high BLAST hit scores [4], [13], [14], indicating that *Yersinia* type III effector inhibitors may function in apoptosis induction. In fact, type III effector inhibitors block the growth of *Waddlia* in host cells, which is one of the primitive chlamydiae [24]. We therefore examined the effect of type III inhibitors (ME0052, ME0053, and ME0054) on the apoptosis using DAPI staining and western blotting. As a result, in contrast to our expectation, no effect of the inhibitors on the apoptosis was observed (Figure S1).

### *Protochlamydia*-secreted CPAF is Involved in the Apoptosis

**Figure 5:**
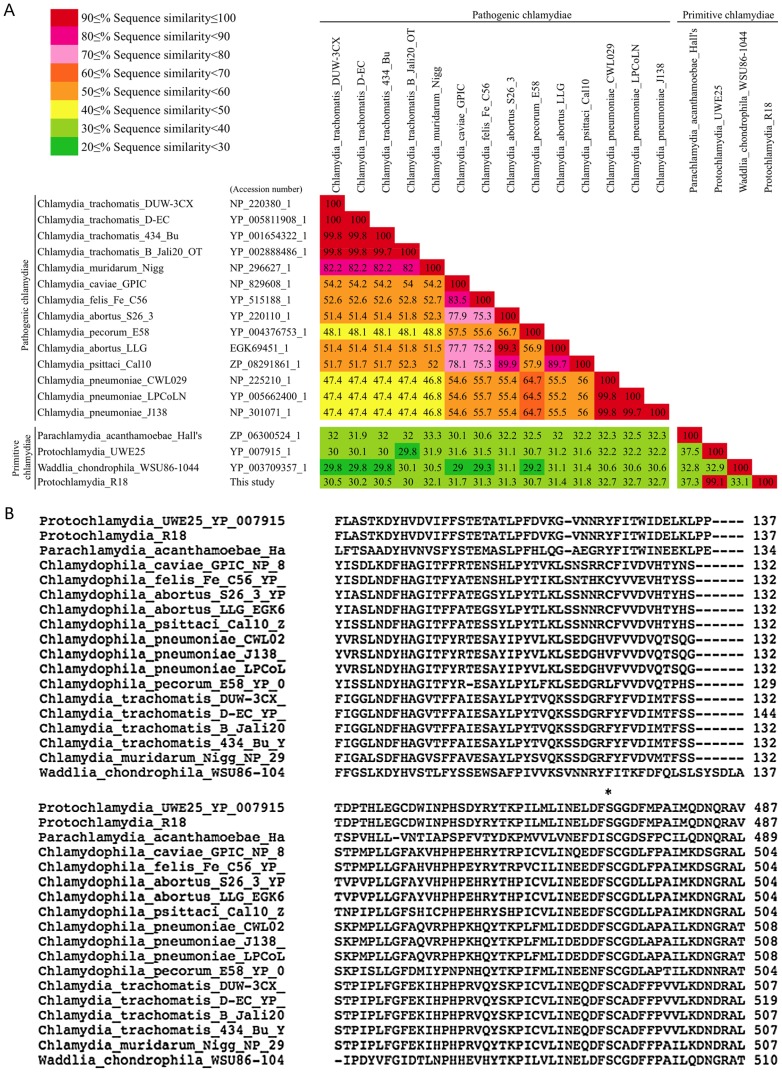
Comparison of similarity scores and conservation of CPAF active centers among CPAFs. A) Comparison of similarity scores among CPAFs. A high score indicates high similarity (Maximum, 100; Minimum 27). Similarity scores among CPAFs were determined by ClustalW2 (See Methods). *accession numbers. B) Alignment of CPAF amino acid sequences. An alignment of CPAFs was constructed by ClustalW2 (See Methods). *critical amino acids, the histidine-101 and serine-499 of CPAF (*C. trachomatis* DUW_3CX), which are in the active center for CPAF activity.

**Figure 6:**
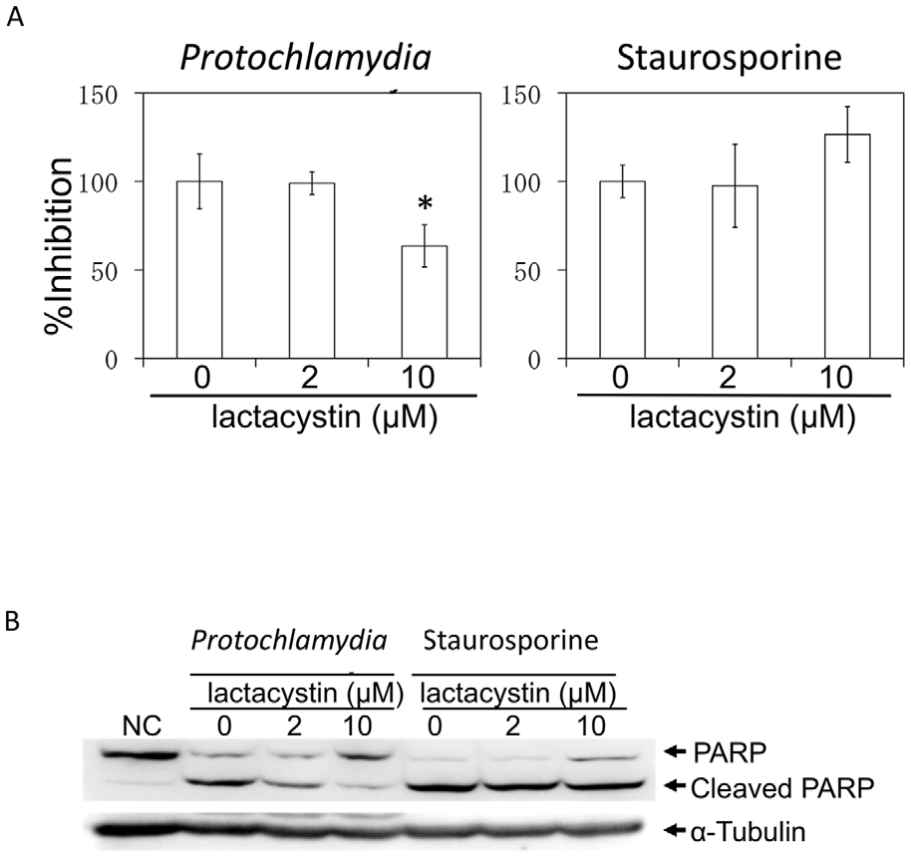
*Protochlamydia* CPAF is involved in apoptosis induction. A) Effect of lactacystin on apoptosis induced by *Protochlamydia*. Cells were cultured with bacteria (MOI 100) in the presence or absence lactacystin (2 or 10 µM) for 24 h. The number of dead cells was estimated by DAPI staining. Data are the means ± SD from at least three independent experiments performed in triplicate. *p<0.05 vs. the absence of lactacystin. B) Representative western blot showing changes of PARP cleavage induced by *Protochlamydia* in the presence of lactacystin or Staurosporine as a control (2 or 10 µM). Cells stimulated with bacteria were collected at 8 h after incubation and then subjected to western blotting with an antibody against PARP. α-tubulin; internal control.

We next assessed whether CPAF is involved in the apoptosis. CPAF was widely conserved among chlamydiae including *Protochlamydia* R18 used in this study, although similarity scores differed among chlamydiae (Figure 5A). Interestingly, while the similarity scores among pathogenic chlamydiae were very high (% of sequence similarity; 47.4–100), the scores among primitive chlamydiae were surprisingly low (% of sequence similarity; 29–100) (Figure 5A). However, histidine-101 and serine-499 in CPAF (*C. trachomatis;* DUW_3CX), which are two critical residues in the active center [25], were well conserved between primitive and pathogenic chlamydial CPAFs (Figure 5B). Thus, the data suggested that primitive chlamydial CPAF from *Protochlamydia* is still active and has a critical role in amoebal and mammalian cells. It is therefore expected that a CPAF inhibitor, lactacystin, which directly binds to and blocks pathological chlamydial CPAF activiy [25], could similarly work for inhibition of primitive chlamydial CPAF activity as well, possibly preventing apoptosis. As a result, lactacystin caused a decrease in the number of dead cells (Figure 6A) and blocked PARP cleavage, which was dependent on drug concentration (Figure 6B), indicating that CPAF was involved in the induction of HEp-2 cell apoptosis. In addition, effect of lactacystin itself on inhibition of apoptosis is minimal because of more like lactacystin acting as an accelerator on apoptosis [26].

## Discussion

It is well known that apoptosis occurs in human cells through two distinct pathways, namely extrinsic and intrinsic pathways [27]–[30]. The extrinsic pathway is induced by physical or environmental stimuli such as TNFα through the cellular death receptor, followed by caspase-8 and −3 activations [28], [29]. The intrinsic pathway begins internal stresses such as accumulation of miss-folded proteins or effector molecules secreted by pathogens, followed by mitochondrial disability, and caspase-9 and −3 activations [30]. Both pathways require PARP cleavage for complete activation, which is located downstream of the apoptosis pathway, and is directly linked to nuclease activation [27]–[30]. Because caspase-9 and −3 activations occur with PARP cleavage, it is clear that the apoptosis pathway induced by *Protochlamydia* is the intrinsic pathway. The finding that another primitive chlamydial strain, *Protochlamydia* UWE25, similarly induces apoptosis of the insect cell line S2 cells by DNA fragmentation [31] supports our data.

As described previously, heat- or UV-killed *Protochlamydia* do not induce apoptosis [22]. In this study, we also confirmed that apoptosis begun at an early time point until almost 8 h after incubation. Moreover, we found that cytochalasin D blocked the apoptosis, indicating a requirement for bacterial entry into cells. It is well known that pathogenic chlamydial effectors manipulate the host response to create an optimal cellular environment [12]–[21]. It is also known that effector proteins are synthesized at a late stage of the developmental cycle [32]–[34], possibly accumulating in the infective *Protochlamydia* progeny to infect host cells as a secondary infection. Type III effectors, which are pathogenic chlamydial Incs, have been well investigated, and are responsible for the process of inclusion biogenesis [35]–[37]. Thus far, primitive chlamydial Incs with similar features to those of pathogenic chlamydiae have been identified [16]. In the present study, *Yersinia* type III inhibitors could not block the apoptosis. However, possible role of type III effectors in such apoptosis still remains unclear. It has been reported that *Protochlamydia* UWE25 possesses approximately 100 Kb of inserted island genes consisting of 72 distinct genes in the genome, which possibly encode type IV secretion machinery [38]. Although our genome sequencing data of *Protochlamydia* R18 also revealed the presence of *Protochlamydia* R18 ORFs against the UWE25 type IV gene cluster with a minimal BLAST hit score (data not shown), possible role of type IV effectors in the apoptosis also remains unknown. Furthermore, pathogenic chlamydiae possess CPAF as mentioned above, which is responsible for inclusion maturation through cellular matrix degradation of the inclusion membrane backbone, and is possibly secreted by type II secretion machinery. This process provides flexibility to mature inclusion bodies, dependent on the bacterial amount, and prevent apoptosis of infected cells through degradation of BH3-only proteins [17]–[21]. Meanwhile, pathogenic chlamydial CPAF appears to have a broad range of substrate specificity and thus it could not deny that the specificity had changed through evolution from ancestral chlamydiae. We therefore focused on type II secretion effector CPAF as an intriguing candidate in the apoptosis induction. In fact, we clearly demonstrated that *Protochlamydia* CPAF is required for the apoptosis induction in HEp-2 cells.

While the amino acid sequence of *Protochlamydia* R18 CPAF was found to have 99.1% (% of sequence similarity) with that of UWE25 CPAF, the R18 CPAF sequence did not resemble pathogenic chlamydial CPAFs [about 31.4% (% of sequence similarity)]. This finding suggests that ancestral chlamydial CPAF drastically changed after diverging into primitive and pathogenic chlamydiae. Moreover, histidine-101 and serine-499 of *C. trachomatis* CPAF, which are in the active center of activity [25], were obviously conserved among chlamydiae, regardless of primitive chlamydiae. This observation also suggests that CPAF activity is very important to achieve adaptation in host cells, and has a critical role in manipulation of host cells.

Interestingly, pathogenic chlamydial infection (*C. trachomatis* L2) blocked the apoptosis (data not shown). It is well known that inhibition of pathogenic chlamydia-induced apoptosis occurs through degradation of BH3-only proteins (Puma, Bim, and Bik) by CPAF secreted into infected cells [17]–[21]. However, other substrates of CPAF may be involved in the apoptosis [39]. Thus, it is possible that pathogenic chlamydial CPAF interacts with primitive chlamydial CPAF itself to block *Protochlamydia*-induced apoptosis, implying a critical role of pathogenic chlamydial CPAF with a wide range of substrate specificities obtained through evolution.

**Figure 7:**
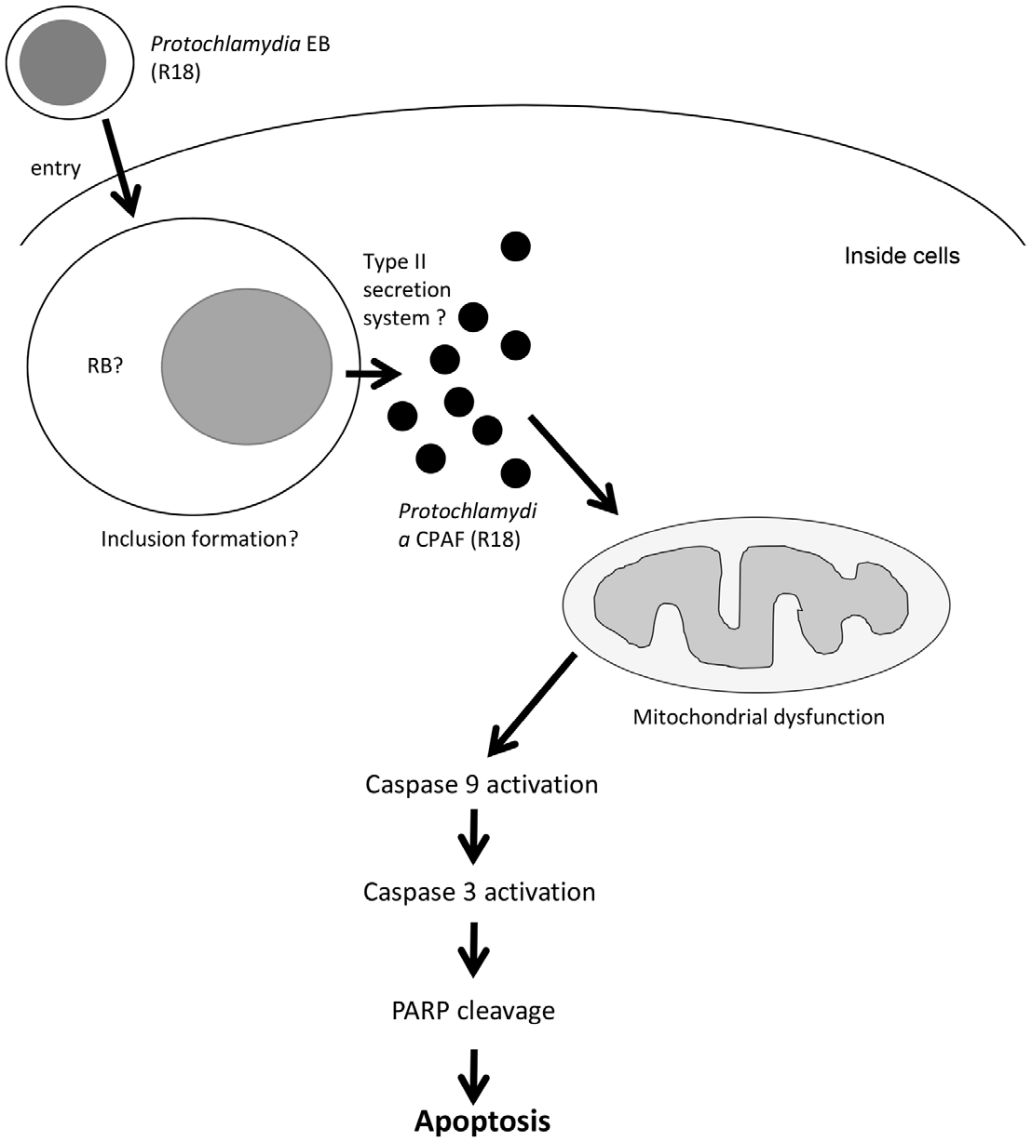
Hypothetical pathway of *Protochlamydia*-induced apoptosis of immortalized human HEp-2 cells.

In conclusion, we elucidated the molecular mechanism of HEp-2 cell apoptosis induced by *Protochlamydia* R18, showing that after entry, *Protochlamydia*-secreted CPAF induces mitochondrial dysfunction with a decrease of the membrane potential, followed by caspase-9, caspase-3 and PARP cleavages for apoptosis (a hypothetical scenario, Figure 7). The finding suggests a possible evolutionary path in which pathogenic chlamydiae may begin to eliminate the strong cytopathic effect of ancestral chlamydia on mammalian cells, and a serine-protease enzyme, CPAF, may be a key molecule that determines the survival of host cell.

## Methods

### Bacterial Stocks and Assessment of Numbers

*Protochlamydia* R18 used in this study was originally isolated from a river in Sapporo, Japan, as an endosymbiont found in environmental *Acanthamoeba* [40]. We also confirmed that the *Protochmaydia* 16S rRNA sequence was identical to that of *Protochlamydia* UWE25 (AB506679) with a 99.2% identity score [40]. The bacterial stock was prepared as follows. Bacteria were maintained within infected amoebae. Briefly, infected cells were harvested and disrupted by freeze/thawing. After centrifugation at 180× *g* for 5 min to remove cell debris, bacteria were concentrated by high-speed centrifugation at 800× *g* for 30 min. The bacterial pellet was resuspended in sucrose-phosphate-glutamic acid buffer consisting of 0.2 M sucrose, 3.8 mM KH_2_PO_4_, 6.7 mM Na_2_HPO_4_ and 5 mM L-glutamic acid (pH 7.4), and then stored at –80°C until needed. The number of EB was determined by an amoeba-infectious unit (AIU) assay, using a co-culture of the amoebae, established previously [41].

### Amoebae and Human Cells

Free-living amoebae, *A. castellanii* C3 (ATCC 50739), were purchased from the ATCC and used to assess bacterial infectious progenies by an AIU assay. As mentioned above, amoebae harboring *Protochlamydia* were used to prepare bacterial stocks. Both amoebae were maintained in PYG broth (0.75% (w/v) peptone, 0.75% (w/v) yeast extract and 1.5% (w/v) glucose) at 30°C [22]. Immortalized epithelial cell line HEp-2 purchased from the Reken Cell Bank (Ibaraki, Japan) was used for assessment of cell death.

### Drugs

Cytochalasin D and lactacystin were purchased from Sigma (St. Louis, MO) and Enzo life sciences (Farmingdale, NY), respectively. Z-VAD-FMK (pan-caspase inhibitor) and other caspase inhibitors [Z-WEHD-FMK (caspase-1 inhibitor), Z-DEVD-FMK (caspase-3 inhibitor), Z-IETD-FMK (caspase-8 inhibitor), and Z-LEHD-FMK (caspase-9 inhibitor)] were purchased from the Peptide Institute (Osaka, Japan) and R&D Systems (Minneapolis, MN). All drugs except lactacystin were dissolved in DMSO according to the manufacturer’s instructions, and stored at −20°C until use; lactacystin was also dissolved in water and stored at −20°C. *Yersinea* type III inhibitors (ME0052, ME0053, and ME0054) were kindly provided by Dr. Mikael Elofsson (Umeå University, Sweden), which were also dissolved in DMSO and stored at room temperature.

### Cell Cultures with Bacteria

Immortalized HEp-2 cells (2×10^5^) were cultured with or without bacteria adjusted to an MOI of 10–100 or with staurosporine (10 µM) (Sigma), as a positive control for induction of apoptosis, for up to 24 h at 37°C with 5% CO_2_ in DMEM containing 10% heat-inactivated fetal calf serum. Cells were also cultured in the presence or absence of bacteria with or without caspase inhibitors (100 µM) (See above), cytochalasin D (0.5–2 µM) or lactacystin (2, 10 µM). No cytotoxicity of these drugs at working concentrations in the cells was confirmed.

### Assessment of Cell Death

Cell death was estimated by changes of nuclear morphology using DAPI staining according to our previous study [22]. Experiments were performed independently at least three times. Data were expressed as the mean ± standard deviation (SD).

### Western Blot Analysis

Cells collected from each culture were boiled for 5 min at 100°C in a reducing sample buffer containing 2-mercaptoethanol. Then, sample was loaded and separated by 10% (w/v) SDS-PAGE (20 mA, 80 min). Separated proteins were transferred to a polyvinylidene difluoride membrane by semi-dry electroblotting. Membranes were blocked with 5% (w/v) skim milk in TBS-T and then incubated with an anti-PARP (Roche Diagnostics, Indianapolis, IN) for 1 h at room temperature, followed by a HRP-conjugated goat anti-rabbit IgG for 1 h at room temperature. Labeled proteins were visualized with Pierce ECL western blotting substrate (Thermo Scientific).

### Assessment of Mitochondrial Membrane Integrity

HEp-2 cells were stimulated with *Protochlamydia* for 8 h, and then incubated with 100 nM MitoTracker Red CMXRos (Invitrogen, Grand Island, NY) for 30 min at 37°C according to the manufacturer’s instructions. After fixation with 4% paraformaldehyde, the cells were observed under a confocal laser scanning microscope.

### Full-length CPAF Amino Acid Sequences and Calculation of Similarity Scores among CPAFs

*Protochlamydia* R18 full-length *cpaf* gene sequence (AB747349) was obtained from *Protochlamydia* R18 draft genome sequence (data not shown). Other CPAF amino acid sequences were also obtained from the NCBI database (http://www.ncbi.nlm.nih.gov/); *Chlamydia trachomatis* DUW-3CX (NP_220380_1), *Chlamydia trachomatis* D-EC (YP_005811908_1), *Chlamydia trachomatis*_434_Bu (YP_001654322_1), *Chlamydia trachomatis* B_Jali20_OT (YP_002888486_1), *Chlamydia muridarum* Nigg (NP_296627_1), *Chlamydophila caviae* GPIC (NP_829608_1), *Chlamydophila felis* Fe_C56 (YP_515188_1), *Chlamydophila abortus* S26_3 (YP_220110_1), *Chlamydophila pecorum*_E58 (YP_004376753_1), *Chlamydophila abortus* LLG (EGK69451_1), *Chlamydophila psittaci* Cal10 (ZP_08291861_1), *Chlamydophila pneumoniae* CWL029 (NP_225210_1), *Chlamydophila pneumoniae* LPCoLN (YP_005662400_1), *Chlamydophila pneumoniae* J138 (NP_301071_1), *Parachlamydia acanthamoebae* Hall’s (ZP_06300524_1), *Protochlamydia* UWE25 (YP_007915_1), and *Waddlia chondrophila* WSU86-1044 (YP_003709357_1). Alignment with % of sequence similarity among CPAFs were constructed by Clustal Omega software (EMBL-EBI; http://www.ebi.ac.uk).

### Statistical Analysis

Comparisons of bacterial numbers were assessed using an unpaired *t*-test. A value of *p*<0.05 was considered significant.

## Supporting Information

Figure S1 Effect of type III inhibitors on apoptosis induced by *Protochlamydia*. A) Cells were cultured with bacteria (MOI 100) in the presence or absence of each type III inhibitor (ME52, ME53, and ME54) for 24 h. The number of dead cells was estimated by DAPI staining. Data are the means ± SD from at least three independent experiments performed in triplicate. B) Representative western blot showing changes of PARP cleavage induced by *Protochlamydia* in the presence of each type III inhibitor (ME52, ME53, and ME54). Cells stimulated with bacteria were collected at 8 h after incubation and then subjected to western blotting with an antibody against PARP. DMSO; solvent control. α-tubulin; internal control.
(TIF)
