# Supplementary figures and images for: Protochlamydia Induces Apoptosis of Human HEp-2 Cells through Mitochondrial Dysfunction Mediated by Chlamydial Protease-Like Activity Factor
Source: PLoS One. 2013 Feb 11;8(2):e56005. doi: 10.1371/journal.pone.0056005 (PMC3569409; doi:10.1371/journal.pone.0056005)

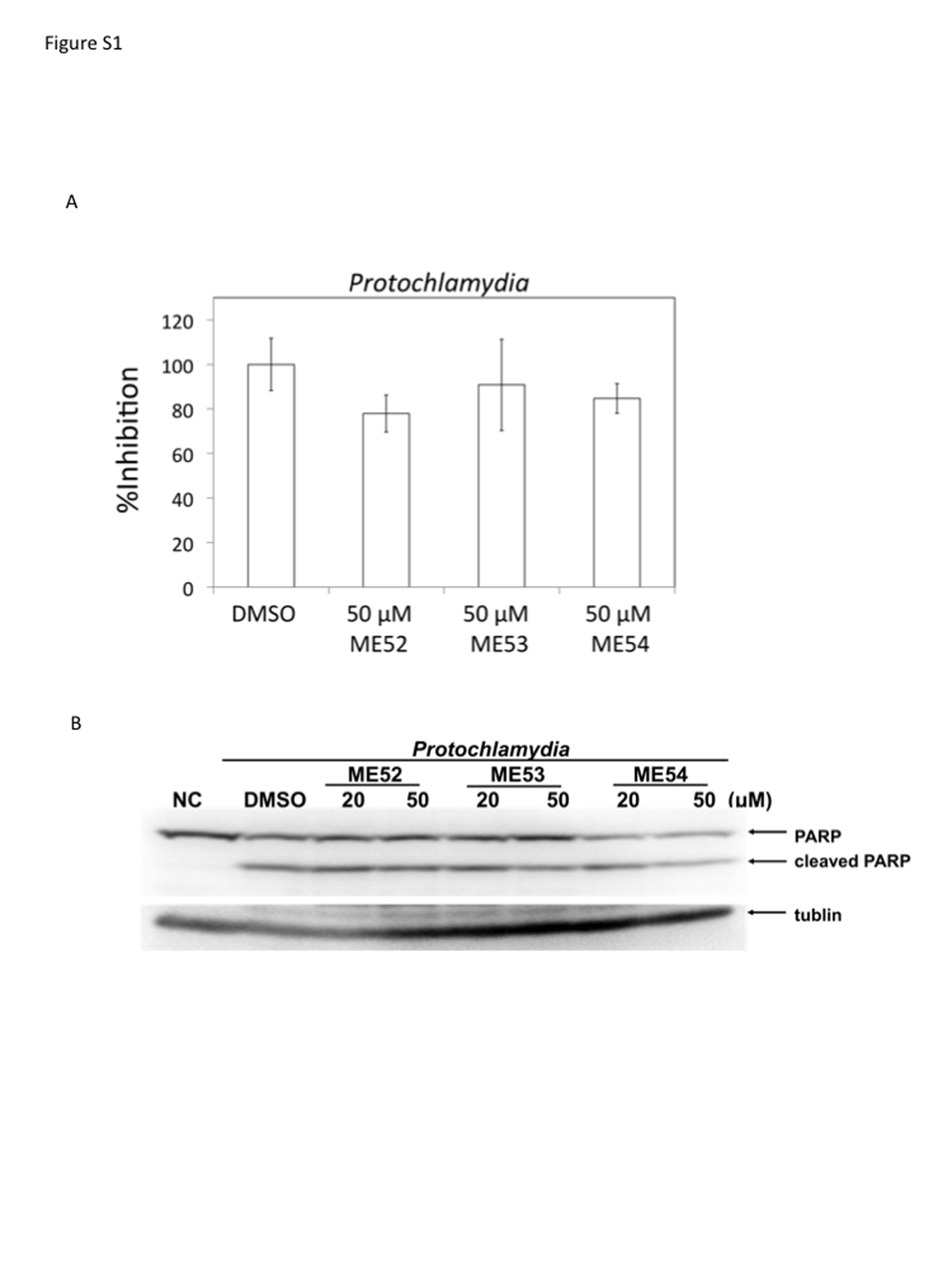

Supplement: Figure S1 — Effect of type III inhibitors on apoptosis induced by Protochlamydia. A) Cells were cultured with bacteria (MOI 100) in the presence or absence of each type III inhibitor (ME52, ME53, and ME54) for 24 h. The number of dead cells was estimated by DAPI staining. Data are the means ± SD from at least three independent experiments performed in triplicate. B) Representative western blot showing changes of PARP cleavage induced by Protochlamydia in the presence of each type III inhibitor (ME52, ME53, and ME54). Cells stimulated with bacteria were collected at 8 h after incubation and then subjected to western blotting with an antibody against PARP. DMSO; solvent control. α-tubulin; internal control. (TIF) [file pone.0056005.s001.tif]
